# Uterine uptake of estrogen and progestogen-based radiotracers in rhesus macaques with endometriosis

**DOI:** 10.21203/rs.3.rs-3311162/v1

**Published:** 2023-09-08

**Authors:** Rachel Catharine Wilson, Jeanne M. Link, Yueh Z. Lee, Jorge D Oldan, Steven L. Young, Ov D Slayden

**Affiliations:** Whitman College; Oregon Health & Science University; The University of North Carolina at Chapel Hill; The University of North Carolina at Chapel Hill; Duke University; Oregon Health & Science University

**Keywords:** Positron emission tomography, FES, FFNP, menstrual cycle

## Abstract

**Purpose:**

Few investigations have examined the uptake of radiotracers that target the prominent sex-steroid receptors in the uterus across the menstrual cycle and with disease state. We aimed to determine if uptake of the radiotracers that target estrogen and progesterone receptors (ER and PR) differ with the presence of endometriosis and/or across the menstrual cycle. We performed PET and computed tomography (CT) imaging procedures on rhesus macaques (*Macaca mulatta*) using 16α-[18F]fluoroestradiol (FES) and 21-[18F]fluoro-furanyl-nor-progesterone (FFNP) in individuals with and without endometriosis in the proliferative and secretory phases of the menstrual cycle.

**Procedures:**

Macaques with either clinically diagnosed endometriosis (n = 6) or no endometriosis (n = 4) underwent abdominopelvic PET/CT scans with FES. A subset of these animals also underwent PET/CT scans with FFNP. Standard uptake values corrected for body weight (SUVbw) were obtained for each radiotracer in target and background tissues (i.e., intestinal and muscle). We performed repeated measure analysis of variance tests to determine how uterine and background uptake differed with scan time, phase of the menstrual cycle, and disease state.

**Results:**

PET/CT could not resolve small, individual endometriotic lesions. However, uterine uptake of both radiotracers was elevated in the proliferative phase compared to the secretory phase of the menstrual cycle. Intestinal uptake exhibited greater variation during the proliferative phase compared to the secretory phase. Further, intestinal uptake of FFNP increases as the scan progresses, but only during the proliferative phase. Muscle uptake did not differ with menstrual phase or radiotracer type. Lastly, macaques with endometriosis displayed higher uterine uptake of FES compared to those without endometriosis.

**Conclusions:**

PET/CT with FES and FFNP support the concept that ER and PR levels are altered in individuals with endometriosis. This highlights the impact of the disease on typical reproductive tract function and may provide a novel pathway for the identification of individuals with endometriosis.

## Introduction

Positron emission tomography (PET) imaging with 16α-[18F]fluoroestradiol (FES) and 21-[18F]fluoro-furanyl-nor-progesterone (FFNP) are validated for imaging of estrogen receptor (ER) and progesterone receptor (PR) responsive diseases, specifically cancer [1]. Cancers are not the only disease in which ER and PR signaling can be disrupted. Various other diseases namely reproductive disorders (e.g., endometriosis, polycystic ovarian syndrome, leiomyomas, etc.) are associated with disrupted ER and PR physiologies [2–3]. Therefore, examining the in vivo presence, absence, abundance, and activity of ER and PR in the reproductive tract using PET imaging and FES/FFNP radiotracers represents an opportunity to provide a more dynamic understanding of how certain disease states may alter ER and PR signaling.

As nuclear steroid receptors, ER and PR are transcription factors that mediate actions of steroid hormones in the reproductive tract and many other organ systems [4–6]. In the reproductive tracts of human and nonhuman primates, estrogen receptors (ER) and progesterone receptors (PR) undergo hormone-dependent regulation during the menstrual cycle [7]. For example, in the uterus, estrogen stimulates expression of endometrial ER and PR while progesterone reduces expression of ER and PR with differential regulation of specific PR isoforms (PRA vs PRB).

In certain diseases, such as endometriosis, progesterone action is attenuated resulting in altered hormone responsiveness in the reproductive tract and in endometriotic tissues [8–9]. This is often referred to as progesterone resistance [10]. Endometriosis is also an estrogen-dependent disorder in which the ectopic endometrium is present outside of the uterus; ectopic as well as eutopic endometrium responds to typical changes in estrogen throughout the menstrual cycle thereby altering ER and PR abundance [11]. Therefore, clinical and research tools developed for hormone dependent cancers such as PET imaging can be applied to endometriosis [12–13].

Our broad premise is that PET imaging of ER and PR with FES and FFNP tracers can provide an invaluable tool for identifying subjects with endometriosis and tracking the progression of the disease. Safety profiles for PET imaging with FES and FFNP of patients with hormone-dependent cancer have been well documented [14]. However, this approach has not been assessed in patients with endometriosis and is not the current “standard of care”. Laparoscopic surgery to identify and stage lesions is currently the only reliable tool for a diagnosis of endometriosis. Moreover, little is known regarding the uptake of these tracers in the reproductive tract during the menstrual cycle, or the impact of endometriosis-induced progesterone resistance on tracer uptake. Therefore, preclinical baseline studies on reproductive tract uptake of these tracers is essential prior to clinical trials using this methodology.

In this study we conducted multiple PET/CT imaging experiments with both FES and FFNP on rhesus macaques (*Macaca mulatta*). Rhesus macaques are menstruating nonhuman primates with approximately 28-day menstrual cycles similar to those of humans [15–16]. Like humans, macaques develop spontaneous endometriosis [17–19]. Additionally, the strengths of the macaque model include: 1) our capability to extensively assess menstrual cycle length and phase by quantifying serum sex-steroid hormones levels and performing daily vaginal swabs [9, 20]; 2) to carryout repeated PET imaging trials that exposes the same animal to repeated radiation; and 3) to be capable of collecting and assessing endometriosis and the reproductive tract after PET imaging. Our primary goal was to assess tracer uptake by the uterus across the menstrual cycle. We further sought to determine if either FES or FFNP provides more specificity and/or spatial resolution in the ability to identify potential lesions due to the preferential uptake of radioligand based on hormone receptor [21].

## Methods

### Animal ethics and welfare

The Institutional Animal Care and Use Committee (IACUC) at the Oregon National Primate Research Center (ONPRC) and Oregon Health & Science University authorized all experiments and procedures following the U.S. Public Health Service Policy on Humane Care and Use of Laboratory Animals [22]. The division of Comparative Medicine/Animal Resources and Research Support at ONPRC oversaw all care of rhesus macaques. Veterinarians identified macaques suspected to have endometriosis by performing ultrasonography. Diagnosis of endometriosis was confirmed by postmorteum visualization of lesions. Laparoscopic examination ensured no lesions were present in controls. The age of control macaques ranged between 9–17 years with an average of 13, whereas macaques with endometriosis ranged from 11–18 years old with an average of 15.

### Radiotracer synthesis and analysis

Radiotracers were prepared by the OHSU Center for Radiochemical Research following previously published procedures for FES [23–24] and FFNP [25–26]. Synthesis of FES was performed using a routine “large format” using a cyclotron. In contrast synthesis of FFNP was found to be less reliable resulting in the need for small batch preparation. Troubleshooting and optimizing procedures for FFNP resulted in failed opportunities to scan monkeys particularly for control animals in the proliferative phase.

**Figure 1:**
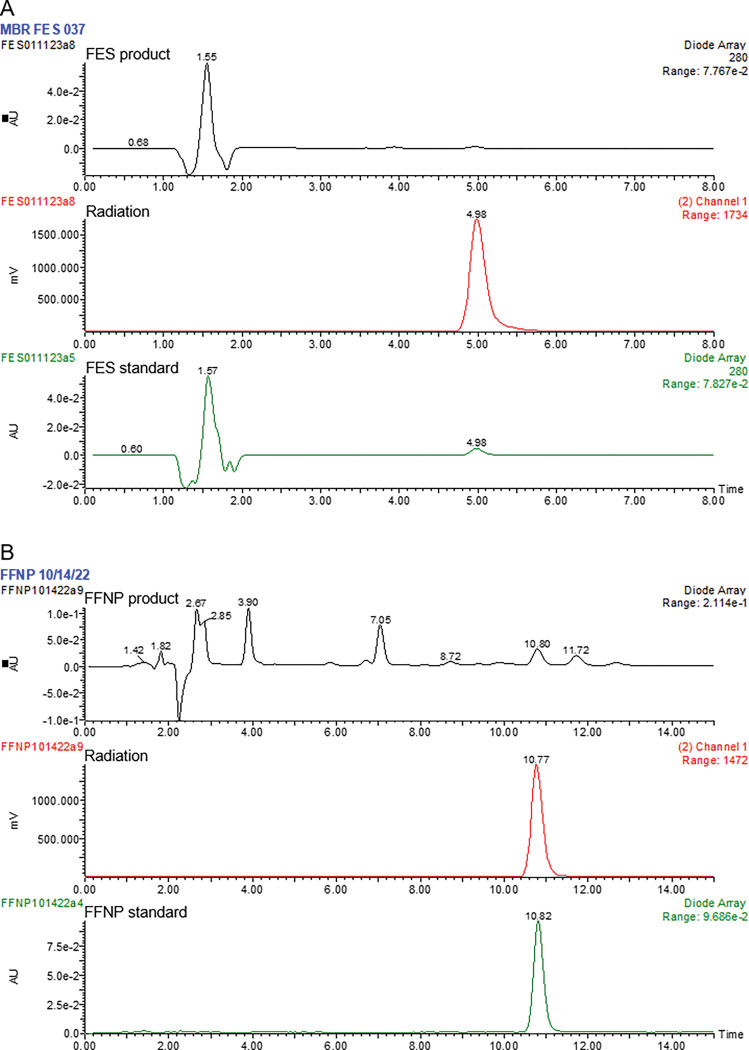
Ultraviolet absorbance and radiation curves for 16-α-18F-fluoro-17-β estradiol (FES; A) and 21–18F-fluorofuranyl-norprogesterone (FFNP; B). A peak with the retention time of the corresponding standard can be observed in both radiotracer products: 5.0 minutes for FES and 10.8 minutes for FES. Small impurities can be observed in the FES and FFNP products, but they are not radioactive.

Immediately after synthesis, high-performance liquid chromatography and mass spectroscopy analyses were conducted to evaluate radiochemical purity and measure molar concentrations. Radiochemical purity was 98% or greater for all FES syntheses, and 92% or greater for all FFNP syntheses with the exception of one (78.9%; Fig. 1). The standard uptake values (SUVs) for the synthesis with lower purity was within the range of SUVs for all other scans. Radioactivity of tracers at injection ranged from 57.0 to MBq with an average of 122.0 ± 3.6 MBq. The average concentration (μg/mL ± standard deviation) for FES was 1.42 ± 1.03 and for FFNP was 1.42 ± 1.01.

### PET/CT scan procedure and analysis

Macaques with and without endometriosis (n = 6 and n = 4, respectively) were fasted overnight, sedated with ketamine, intubated, and anesthetized with isofluorane. We placed arterial, venous, and urinary catheters for the collection of serial blood samples, injection of radiotracers, and to promote the passive emptying of the urinary bladder. Animals were placed in dorsal recumbency after anesthetization. The CT was performed prior to the PET scan using a Discovery 610 Scanner (GE Healthcare, Boston, MA).

The PET scan was started 2.5 minutes prior to injection of radiotracers. Images from PET scans were obtained for 1.5 hours. Acquisition rates were as follows: 60 frames at 5 sec/frame, 8 frames at 15 sec/frame, 4 frames at 30sec/frame, 3 frames at 60 sec/frame, and 40 frames at 120 sec/frame. DICOM images were analyzed using the imaging software MIM (version 7.0.6, Cleveland Ohio). We identified regions of interest (ROIs) across multiple sections using the 2D contouring tool to obtain SUVs accounting for body weight (SUVbw). Contours for the uterus ROI encompassed the entire uterus, while contours for the intestinal ROIs started 15 frames superior to the uterus and included 10 frames of the abdomen excluding muscle tissue. To gain an idea of how uterine uptake compares to other organs, we also identified intestinal and muscle (i.e., psoas major) ROIs to quantify background radiotracer uptake. Evaluating uptake in two different ROIs helped to account for the excretion of FES and FFNP metabolites through the intestine [27]. Maximum SUVbw were exported for background and uterine ROIs. We also obtained average SUVbw and ROI volumes for the uterus. The number of scans were limited due to 1) the number of macaques suspected to have endometriosis, 2) scheduling constraints of coordinating radiotracer production with PET machine availability, and 3) optimizing and troubleshooting FFNP production.

### Statistics

All statistical analyses were performed in SPSS (version 29, IBM SPSS Statistics, Chicago, IL). Any data not adhering to assumptions of statistical tests were log and/or square root transformed.

We initially utilized repeated measure analysis of covariance (RMANCOVA) tests with ligand concentration as a covariate to inform how FES and FFNP uptake may have differed across the menstrual cycle. However, we removed this covariate after finding no significant relationship between radioligand concentration and SUVbw (Table 1). Because we were interested in documenting whether there was a significant difference in FES versus FFNP uptake did not separate statistical analyses based on radiotracer type. Therefore, we performed repeated measure analysis of variance tests to examine how maximal uterine and background SUVbw differ with the following main effects: radiotracer type (between-subjects factors), phase of the menstrual cycle (between-subjects factors), duration of the scan (within-subjects factor). We also investigated interactions among all the main effects and thus present data on how SUVbw may be affected by duration of scan and radiotracer type, duration of scan and phase of menstrual cycle, and finally duration of scan*radiotracer type*phase of menstrual cycle. To account for the likely delay between radiotracer injection and start of scan in a clinical setting we analyzed data from a subset of the scan: 10–90 minutes.

To determine if the presence of endometriosis was associated with differences in altered sex-steroid signaling in the uterus we again used RMANCOVA tests. We compared individuals with and without endometriosis and radiotracer type (between-subjects factors) to determine if uterine uptake levels differed across the duration of the scan (within-subjects factor). We included uterine volume, and initially ligand concentration as covariates. However, similar to maximal uptake levels, we subsequently removed ligand concentration as a covariate because no significant relationships were found (Table 1). We focused our analyses on the secretory phase because of increased variation and lower sample sizes particularly for controls injected with FFNP during the proliferative phase (Figs. 4&5).

## Results

**Figure 2:**
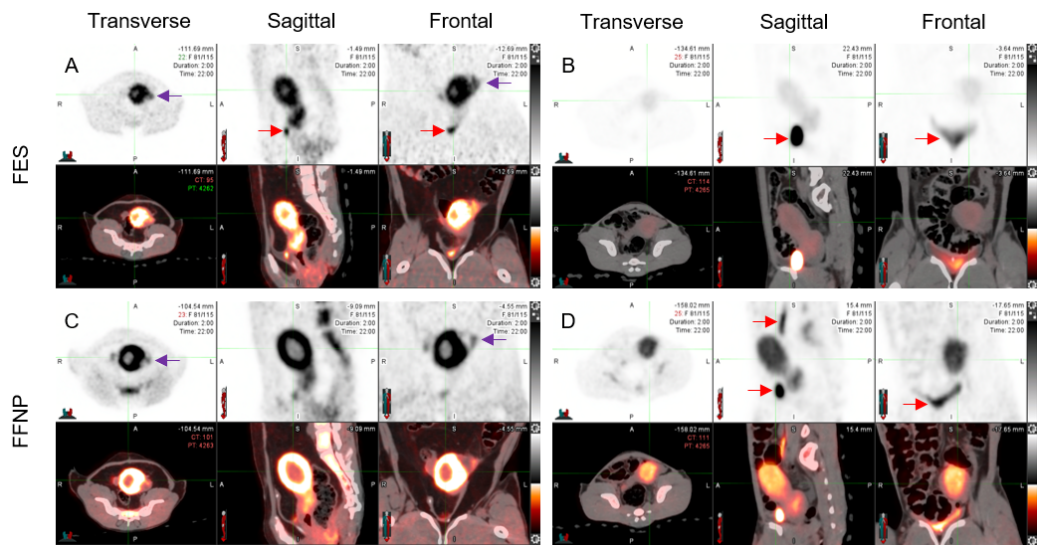
Radiotracer uptake during the secretory phase in rhesus macaques (*Macaca mulatta*) with endometriosis (A&C) and without endometriosis (B&D). Radiotracers target the estrogen receptor (16-α-18F-fluoro-17-β estradiol; FES; Panels A&B) and progesterone receptors (21–18F-fluorofuranyl-norprogesterone; FFNP; Panels C&D). The top panel is the positron emission tomography (PET) scan and the bottom panel is the PET overlay on the computed tomography scan. Purple arrows indicate uptake in an ovarian adhesion that was confirmed upon postmortem dissection (Figure 3). Red arrows indicate radioactivity in the urinary system.

**Figure 3:**
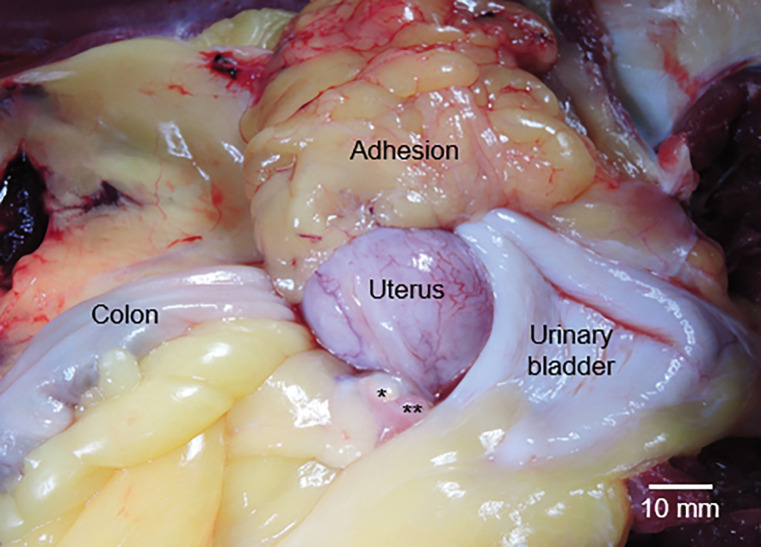
Reproductive tract of the rhesus macaque (*Macaca mulatta*) with endometriosis whose scans are presented in Figure 1A&C. The right ovary indicated by *; the right fimbria with **. We dissected the left ovary and endometriotic lesions out of the adipose adhesion (indicated).

For macaques with endometriosis, we were unable to identify smaller endometriotic lesions because post-mortem examination of the reproductive tract revealed that the majority of lesions were present on the serosa of the uterus, and uterine uptake occluded lesion uptake. However, we were able to observe FES and FFNP uptake in atypical soft tissue surrounding the uterus (Fig. 2). We subsequently found endometriotic lesions in adipose tissue adhered to the uterus and ovary (Fig. 3).

Anecdotally, it was difficult to distinguish the myometrium from the endometrium using just CT. With overlaying the PET scan, the highest uptake levels of radiotracers in the field of view of the scan can be observed in the uterus (Fig. 2). Within the uterus there is more uptake of radiotracers in the myometrium compared to the endometrium. Other reproductive structures such as the ovary and cervix also display uptake of radiotracers. Accumulation of excreted radiotracers both FES and FFNP can be observed in the urinary system: bladder and ureters (Fig. 2). For FFNP specifically, there was skeletal uptake (Fig. 2).

### Maximum uterine and background uptake levels

**Figure 4:**
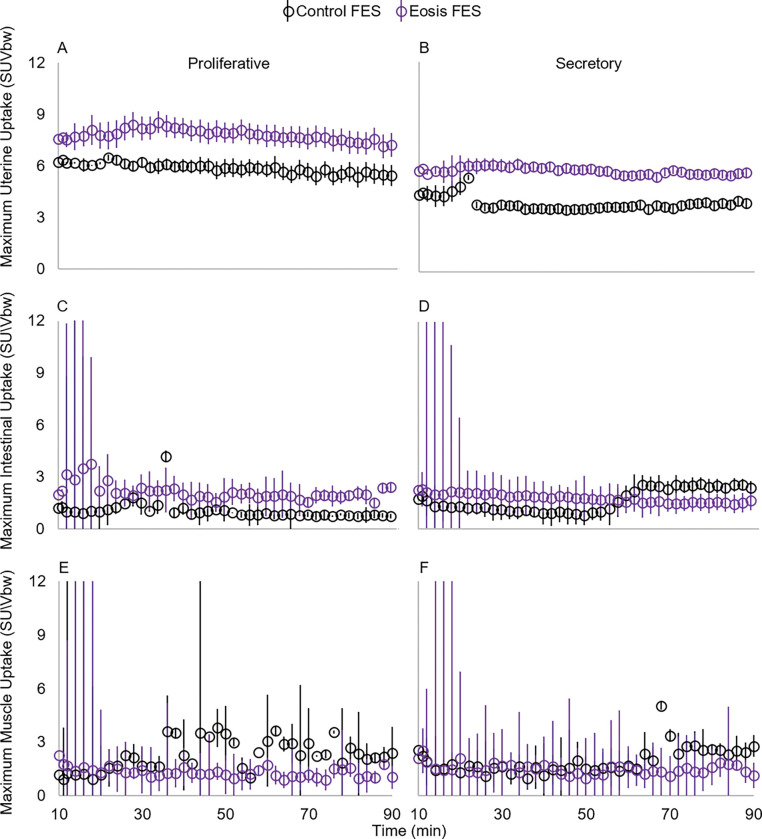
Maximum standard uptake values by body weight (SUVbw) of 16-α-18F-fluoro-17-β estradiol (FES) in the uterus (A&B) and background tissues (C-F) in rhesus macaques (*Macaca mulatta*). Data represent means with standard error bars. Positron emission tomography (PET) scans were performed with computed tomography in the proliferative (left panel) and secretory (right panel) phases of the menstrual cycle. Although presented separately here, we did not include disease state in these statistical analyses for two reasons: maximum SUvbw data was used to determine if there are optimal imaging conditions for identifying individuals with endometriosis and a lack of statistical power due to low sample sizes. A total of 10 macaques were scanned: 6 with endometriosis (Eosis) and 4 without (Controls).

**Figure 5:**
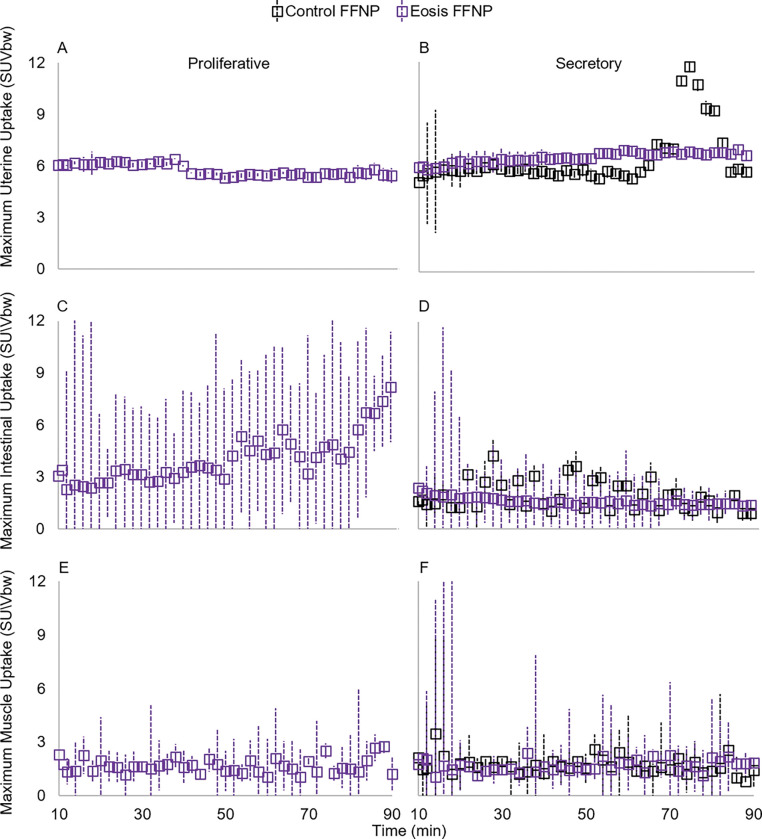
Maximum standard uptake values by body weight (SUVbw) of 21–18F-fluorofuranyl-norprogesterone (FFNP) in the uterus (A&B) and background tissues (C-F) in rhesus macaques (*Macaca mulatta*). Data represent means with standard error bars. Positron emission tomography scans were performed with computed tomography in the proliferative (A&C) and secretory (B&D) phases of the menstrual cycle. Although presented separately here, we did not include disease state in these statistical analyses for two reasons: maximum SUvbw data was used to determine if there are optimal imaging conditions for identifying individuals with endometriosis and a lack of statistical power due to low sample sizes. A total of 10 macaques were scanned: 6 with endometriosis (Eosis) and 4 without (Controls). No controls were scanned in the proliferative phase with FFNP.

Maximum uterine uptake levels significantly varied with time*radiotracer and time*menstrual cycle phase (Table 2; Fig. 4A&B; Fig. 5A&B). These relationships are likely driven by the increase in FFNP uptake between 70 and 90 minutes of scan time, and only during the secretory phase of the menstrual cycle. No between-subject factors were significant (Table 2).

Maximum intestinal uptake levels significantly varied with time*radiotracer, time*menstrual cycle phase, and time*radiotracer*menstrual cycle phase (Table 2; Fig. 4C&D; Fig. 5C&D). The significant interaction of time*radiotracer*menstrual cycle is likely driven by 1) the increase in FES at 60 minutes during secretion (Fig. 4C&D), and 2) the increase of FFNP with scan time in the proliferative phase (Fig. 5C&D). We found a significant between-subjects factor of menstrual cycle phase on maximum intestinal uptake and a near significant effect of radiotracer (Table 2) with increased uptake during the proliferative phase (Fig. 4C&D and Fig. 5C&D).

Maximum muscle uptake did not significantly differ with any within- or between-subjects factors (Table 2; Fig. 4E&F; Fig. 5E&F).

### Average uterine uptake levels between macaques with and without endometriosis

**Figure 6:**
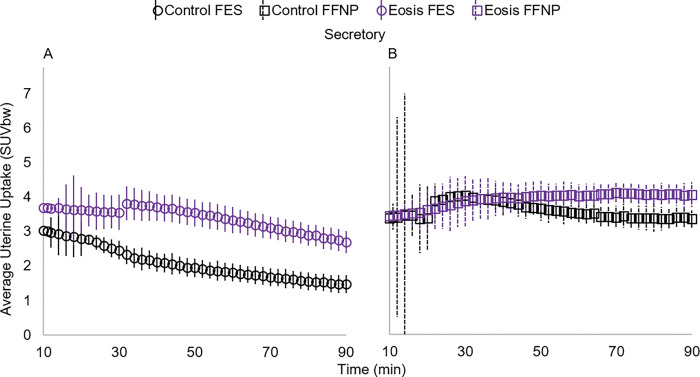
Average uterine standard uptake values by body weight (SUVbw) during the secretory phase in rhesus macaques (*Macaca mulatta*) with and without endometriosis [Eosis (n=6), Controls (n=4)]. Positron emission tomography scans were performed with 16-α-18F-fluoro-17-β estradiol (FES; A) and 21–18F-fluorofuranyl-norprogesterone (FFNP; B).

During the secretory phase we found the within-subjects effects of time, time*uterine volume, time*radiotracer, and time*presence of endometriosis*radiotracer significantly affected average uterine uptake levels (Table 2, Figs. 2&6). We also found a significant between-subjects effect of radiotracer on average uterine uptake with FFNP displaying significantly higher uptake levels compared to FES (Table 2; Figs. 2&6).

## Discussion

Our data show that standard uptake value in rhesus macaques is influenced by radiotracer type, phase of the menstrual cycle, and the presence of endometriosis. Our data support the premise that ER and PR binding is altered in individuals with endometriosis with the finding that average uterine uptake is elevated compared to controls. As such investigations concerning the dynamic signaling of ER and PR may reveal potential etiologies for endometriosis. Focusing on the difference in uptake due to radiotracer and menstrual cycle phase, we suggest that scanning individuals during the secretory phase would provide a higher probability of detecting endometriotic lesions because intestinal background uptake is higher and more variable in the proliferative phase.

Warranting further investigation is that uterine uptake is significantly altered with scan time, radiotracer type, and menstrual cycle. Maximum uterine uptake of both FES and FFNP are relatively constant regardless of menstrual cycle phase up until 70 minutes of scan time. At 70 minutes we observe a substantial increase in uterine FFNP uptake and only during the secretory phase. While we did not statistically account for the presence of endometriotic lesions in the maximum uptake analyses, the increase in FFNP after 70 minutes during the secretory phase is only observed in individuals without endometriosis. The increase is transient and returns back to ‘baseline’ by the end of the scan. The dynamic change in FFNP at the end of secretory phase scans and the increase in background uptake of FFNP after 50 minutes of scan time during proliferation could be explained by either a change in uterine vascular flow and/or the uptake of FFNP metabolites [28]. Determining the physiological and/or cellular basis of FFNP uptake may elucidate potential underlying mechanisms that differ with the disease state of endometriosis.

Our FES results align with previous findings in humans without gynecological disease [29]. That study found that FES uptake was higher in the endometrium during the proliferative rather than the secretory phase [29]. From these results, we can infer that the differences in FES uptake we found between the proliferative and secretory phase were due to differences in the endometrium. Our findings of higher FES uptake in proliferative phase of the menstrual cycle compared to the secretory phase was consistent with endometriotic disease status. However, individuals with endometriosis displayed higher levels of FES uptake compared to individuals without endometriosis.

Unfortunately, the conclusions drawn from any maximum SUVbw data are preliminary as we were unable to perform FFNP scans on individuals without endometriosis during the proliferative phase. However, we used these data to inform whether certain parameters would provide optimal scanning conditions (i.e., scan time, phase of the menstrual cycle, or radiotracer target). We deemed that scanning individuals during the secretory phase would likely allow for the easier detection of individuals with endometriosis because background uptake was more variable during the proliferative phase of the menstrual cycle in all individuals scanned. We subsequently compared average uterine uptake levels during the secretory phase of the menstrual cycle to determine if uptake levels differed with radiotracer type or with disease state.

We found that uterine uptake levels were altered with the presence of endometriotic lesions during the secretory phase with elevated uptake in individuals with endometriosis. Macaques with endometriosis display increased uterine uptake of both FFNP and FES compared to macaques without endometriosis. However, the timing and magnitude of uptake differs with radioligand. Levels of FES and FFNP are relatively constant until 30 minutes into the scan. At this point the uptake of FES in macaques with endometriosis becomes more disparate from those without endometriosis. For FFNP this difference is less prominent than FES and doesn’t occur until around 60 minutes. The observation that uterine uptake levels are different with disease state may represent an alternative approach to a diagnosis for endometriosis using PET imaging, and also supports a pathophysiological difference in estrogen and progesterone signaling.

Breakdown of radiotracers to metabolites likely contributed to the variation in intestinal uptake we observed. This is likely more prominent for FFNP compared to FES with the increased number of metabolites for FFNP. Prior research suggests that FES clearance is rapid and metabolites include glucuronoide and sulfate which are excreted from through the urinary system [30]. Background activity of FES is relatively constant, a finding that our results corroborate [30]. Metabolism and clearance of FFNP occurs in the liver which likely explains the increase in intestinal uptake we observed in FFNP over the course of the scan due to the liver’s role as an accessory digestive organ [31]. FFNP also has the ability to bind to glucocorticoid receptors, which may represent an alternative pathway for the increased intestinal uptake of FFNP we observed [26]. Unfortunately, metabolites have yet to be documented for FFNP.

The ability to identify small endometriotic lesions using this PET/CT procedure remains equivocal as we were unable to detect endometriotic lesions in animals that were later verified to have endometriosis at necropsy. The locality of the lesions made it difficult to delineate endometriotic tissue from reproductive organs. For the vast majority of macaques clinically diagnosed with endometriosis we found small cystic lesions and ectoptic endometrium on the serosa of the uterus or in adipose tissue adhered to reproductive structures. The high uptake of FES and FFNP in the myometrium occluded the potential to observe these small lesions. Ongoing studies in humans and macaques will help determine the efficacy in identifying smaller lesions. Our human study counterpart utilizes magnetic resonance imaging, which may allow for the identification of lesions located proximal to the uterus because of the ability of MRI to anatomically distinguish the various layers of the uterus. And our induced macaque model of endometriosis will help determine if we can detect lesions more distally located to the uterus [32].

The results presented here will likely inform optimal imaging procedures for future scans in primates both human and nonhuman. We posit that the following conditions will likely provide the optimal chances of identifying ectopic endometrium. These parameters culminate from identifying times during the scans where variation of uptake is least for maximum SUVs. Because intestinal uptake is higher during proliferation, we suggest that scans be performed during the secretory phase of the menstrual cycle. The various association among the menstrual cycle, the gastrointestinal tract, and estradiol supports our findings of menstrual cycle phase influencing intestinal uptake [33–35]. Second, if using FES the scanning window should include 25–50 minutes post-injection. Or if using FFNP the scanning window should include 70–90 post-injection.

Although we were unable to identify small endometriotic lesions using this PET procedure, we were able to detect alterations in sex-steroid signaling that differed between individuals with and without endometriotic disease. Our data indicate that performing PET/CT with radiotracers associated with sex-steroid hormones represents a potential alternative approach to a diagnosis of endometriosis. Ongoing studies in humans and in an induced endometriosis model using macaques will further inform the efficiency in using PET as a diagnostic tool. Because disrupted estrogen and progesterone signaling is associated with infertility and other gynecological disorders [e.g., adenomyosis, leiomyoma, polycystic ovary syndrome [2]], we also posit that performing PET scans with FES and FFNP tracers will likely reveal similar and disparate estrogen and progesterone signaling pathways that may allow for targeted treatment.

## Figures and Tables

**Table 1:** Preliminary statistical analyses showing that radioligand concentration does not influence uterine or intestinal/background standard uptake values. This is the rationale for removing ligand concentration as a covariate in the statistical analyses presented in this study. FES: 16-α-18F-fluoro-17-β estradiol. FFNP: 21-18F-fluorofuranyl-norprogesterone.

Dependent variable	Subjects factor		F_(1,41)_ statistic	P-value
Maximal uterine uptake	Within	Time*FES/FFNP concentration	0.08	1.000
	Between	FES/FFNP concentration	0.48	0.505
Maximal intestinal uptake	Within	Time*FES/FFNP concentration	0.91	0.639
	Between	FES/FFNP concentration	0.57	0.462
Maximal muscle uptake	Within	Time*FES/FFNP concentration	0.31	1.000
	Between	FES/FFNP concentration	0.17	0.687
Mean uterine uptake	Within	Time*FES/FFNP concentration	0.04	1.000
	Between	FES/FFNP concentration	0.55	0.548

**Table 2:** Statistical analyses from repeated measure analyses of covariance indicates standard uptake values (SUVs) in the uterus and intestines differ with duration of scan (time), radiotracer type, and phase of menstrual cycle. We also examined if the presence of endometriosis altered average uterine uptake. Italic and bolded text indicate p < 0.05, italic text indicates p < 0.10.

Dependent variable	Subjects factor		F_(1,41)_ statistic	P-value
Maximal uterine uptake	Within	Time	0.61	0.975
		Time*Radiotracer	**2.74**	**< 0.001**
		Time*Menstrual cycle phase	**1.44**	**0.039**
		Time*Radiotracer*Menstrual cycle phase	0.59	0.981
	Between	Radiotracer	0.02	0.893
		Menstrual cycle phase	0.27	0.609
		Radiotracer*Menstrual cycle phase	2.23	0.153
Maximal intestinal uptake	Within	Time	0.97	0.525
		Time*Radiotracer	**1.49**	**0.027**
		Time*Menstrual cycle phase	**2.04**	**< 0.001**
		Time*Radiotracer*Menstrual cycle phase	**1.72**	**0.004**
	Between	*Radiotracer*	*3.81*	*0.063*
		**Menstrual cycle phase**	**4.51**	**0.045**
		Radiotracer*Menstrual cycle phase	1.52	0.230
Maximal muscle uptake	Within	Time	0.66	0.953
		Time*Radiotracer	0.44	0.999
		Time*Menstrual cycle phase	0.56	0.989
		Time*Radiotracer*Menstrual cycle phase	0.61	0.974
	Between	Radiotracer	0.01	0.912
		Menstrual cycle phase	0.09	0.773
		Radiotracer*Menstrual cycle phase	0.03	0.867
Mean uterine uptake *(secretory only)*	Within	**Time**	**5.87**	**< 0.001**
		Time*Uterine volume	**1.63**	**0.012**
		Time*Presence of endometriosis	0.24	1.000
		Time*Radiotracer	**5.87**	**< 0.001**
		Time*Presence of endometriosis*Radiotracer	**1.92**	**0.001**
	Between	Uterine volume	3.45	0.106
		Presence of endometriosis	0.87	0.383
		**Radiotracer**	**145.23**	**0.036**
		Presence of endometriosis*Radiotracer	1.53	0.257
